# Effects of Astaxanthin on the Physiological State of Porcine Ovarian Granulose Cells Cultured In Vitro

**DOI:** 10.3390/antiox13101185

**Published:** 2024-09-30

**Authors:** Xiaofen Yang, Dongping Zhou, Lv Gao, Yanxin Wang, Yun Wang, Ruru Jia, Yuwei Bai, Deshun Shi, Fenghua Lu

**Affiliations:** Guangxi Key Laboratory of Animal Breeding & Disease Control and Prevention, Guangxi University, 75 Xiuling Road, Nanning 530005, China; 2218401004@st.gxu.edu.cn (X.Y.); 2218301049@st.gxu.edu.cn (D.Z.); 2018391015@st.gxu.edu.cn (L.G.); 2018401007@st.gxu.edu.cn (Y.W.); 2218301032@alu.gxu.edu.cn (Y.W.); 2218301009@st.gxu.edu.cn (R.J.); 2318301001@st.gxu.edu.cn (Y.B.); ardsshi@gxu.edu.cn (D.S.)

**Keywords:** porcine ovarian granulosa cells, astaxanthin, in vitro, physiological state

## Abstract

The physiological state of Granulosa cells (GCs) is intricately linked to the growth and development of oocytes. Oxidative stress has been found to cause damage to GCs in vitro. Astaxanthin (AST), a well-known natural ketone-type carotenoid, has demonstrated strong antioxidant properties. This study investigates the impact of astaxanthin supplementation on the physiological state of porcine ovarian granulosa cells cultured in vitro. Variations in morphology, apoptosis, reactive oxygen species (ROS) levels, and the expression of apoptosis and anti-oxidation-related genes in porcine GCs from different passages were observed. Significant morphological changes, increases in apoptosis, and decreases in antioxidant capacity resulting from passage were observed. Subsequently, treatment with 5 μmol/L astaxanthin significantly enhanced cell viability, proliferation, antioxidant capacity and mitochondrial function while also regulating the estradiol (E2) and progesterone (P4) levels. Additionally, the gene expression of antioxidation, E2, and P4 synthesis markers was assessed, revealing reduced apoptosis and ROS levels in porcine GCs. In conclusion, supplementation with 5 μmol/L astaxanthin in vitro effectively enhances the physiological condition of porcine GCs and optimizes the culture system for these cells in vitro. Optimizing the culture system of porcine GCs in vitro can simulate the function of granulosa cells in vivo and provide a theoretical reference for further promoting follicular development, which is beneficial to improving sow fertility in actual production.

## 1. Introduction

Improving the fertility of sows is a key point in the economic benefits of pig farms and the development of the pig industry. Follicular development plays an important factor in sow fertility [1]. The oocyte and companion ovarian granulosa cells (GCs), which form the largest cell group in the ovarian follicle, maintain a close association essential for oocyte competence, luteinization, and ovulation [2]. The main functions of GCs include steroidogenesis, gonadotropin synthesis and secretion, and support for oocyte development and follicular growth [3,4]. It has become evident that the proliferation and differentiation of GCs are the basic physiological functions that support follicular development. GCCs differentiate into different types of granulosa cells during folliculogenesis (such as the theca cell, granulosa cell, cumulus cell, and oocyte-cumulus-corona cell), and different granulosa cells perform different biological functions at different stages to provide support for follicular development [5,6].

There is a good deal of evidence indicating that the transformation from antral follicles to mature follicles requires the proliferation of GCs, including several key factors [7]. Other evidence indicates that the important biological function of GCs is estrogen synthesis. Estradiol (E2) is the main form of estrogen, which is essential for the estrous cycle and ovarian follicular development [8,9]. The cumulus-oocyte-complexes (COCs) formed by GCs and oocytes regulate the communication between oocytes and GCs, with a direct impact on gene expression and protein synthesis, which protects oocytes from the influence of the microenvironment [10,11]. At the same time, GCs provide oocytes with substances such as glucose, cholesterol, and amino acids that cannot synthesize themselves, thereby promoting the maturation of oocytes [12]. Thus, follicular development and the maturation of oocytes cannot happen without the biological functions of GCs. In order to promote the production of dominant follicles and the maturation of oocytes, the culture system of Porcine GCs in vitro helps to simulate the function of granulosa cells in vivo.

Some research proves that damage to GCs function caused by environmental or physiological stress can disrupt ovarian activity and oocyte development [13]. Current studies have confirmed that cells cultured in vitro may be exposed to various stressors that affect the physiological state of GCs, resulting in cellular oxidative damage [14,15]. These stressors include medium temperature, PH, oxygen, medium components, and toxins. Excessive amounts of reactive oxygen species (ROS) and insufficient antioxidant capacity can cause damage to DNA, lipids, and proteins, adversely affecting cell growth and development [16]. Both enzymatic and non-enzymatic antioxidants can scavenge excess ROS. Oxidative stress easily causes mitochondrial dysfunction, cell apoptosis, inflammation, and other problems [17]. How to balance the oxidant and anoxidant systems is of crucial scientific significance in improving cell biological functions and promoting cell growth and development.

The unique structure of natural astaxanthin (AST), a carotenoid belonging to the xanthophyll class, has a super antioxidant capacity, known as “super vitamin E”, and has broad application prospects in human disease and animal production. [18,19]. The antioxidant activity of carotenoids works by directly scavenging ROS and regulating the expression of antioxidant proteins [20]. Studies have revealed that the combinative administration of astaxanthin and Metformin can effectively reduce the apoptosis of mouse granulosa cells and increase ROS levels [21]. Additionally, evidence suggests that astaxanthin can protect the mitochondrial REDOX equilibrium to maintain mitochondrial function, prevent mitochondrial membrane potential loss, increase oxygen consumption, and protect against oxidative stress [22]. Astaxanthin is a super antioxidant, and we hypothesized that astaxanthin supplementation would positively protect cells cultured in vitro and avoid endogenous oxygen-free radical damage.

This study aimed to explore the effect of astaxanthin on the physiological state of porcine ovarian GCs cultured in vitro. We also aimed to optimize the culture efficiency of porcine GCs and provide a theoretical basis for improving the culture system of Porcine GCs in vitro.

## 2. Materials and Methods

### 2.1. Isolation and Culture of Porcine GCs and Porcine GCs Were Treated with AST

Sow ovaries obtained from local slaughterhouses were placed in a prepared beaker filled with 37 °C saline and transported to the laboratory within two hours. A 1 L beaker was prepared with 75% alcohol, into which the ovaries were quickly submerged for surface cleaning and disinfection (this step should not exceed 30 s). Subsequently, the porcine ovaries were cleaned three times with 37 °C normal saline and placed on the 37 °C hot table. Then, using a disposable 10 mL syringe with a 12-gauge needle, the follicular fluid of the follicle size of 3–6 mm was extracted, the oocytes were picked out, and the remaining follicular fluid was collected using a 50 mL centrifuge tube. Filtering was carried out with a 0.45 μm cell screen to remove excess impurities. The filtered follicle fluid was centrifuged at 1200 rpm at room temperature for 3 min to remove the supernatant, and 1 × PBS suspension cell precipitate containing Penicillin-Streptomycin Solution was centrifuged at 1200 rpm at room temperature for 3 min to remove the supernatant; this process was repeated two to three times. Finally, the cells were resuspended in DMEM medium (high glucose-DMEM supplemented with 0.06 mg/mL Penicillin, 0.1 mg/mL Streptomycin, and 10% FBS) containing bispecific antibodies, seeded into cell culture dishes (JET, BIOFIL), and cultured at 37 °C, 5% CO_2_, and fully saturated humidity in an incubator.

Astaxanthin (Sigma, St. Louis, MO, USA, SML0982) was diluted according to the instructions. We used DMSO not exceeding 1/1000 of the total volume of the medium to prepare astaxanthin as a 10 mol/L concentrated storage solution. For the required concentration in subsequent experiments, the concentrated storage solution was added to the DMEM medium for dilution. Thereby, different astaxanthin concentrations (0, 5, 10, 20, 50 μmol/L) were used during porcine GCs culture in vitro. When the cell density reached more than 90%, the following experiment was conducted.

### 2.2. Immunofluorescent Staining

First, 4% paraformaldehyde was added to the treated porcine GCs, and they were placed at room temperature to fix for 20 min and then washed three times for 5 min each in TPBS supplemented with 0.01% Triton X-100 and 0.3% BSA. Then, the porcine GCs were permeabilized for 20 min in PBS supplemented with 1% Triton X-100 and closed in 1% BSA (formulated using PBS) for 1 h; after washing three times for 5 min each time in TPBS, the cells were incubated with the antibodies of anti-FSHR (Wanleibio, Shenyang, China) at 4 °C overnight. On the second day, the porcine GCs were removed from 4 °C and restored to room temperature for 20 min. After that, the cells were incubated with Goat Anti-Rabbit IgG H&L (HRP) (ABclonal, Wuhan, China) for 2 h at room temperature. Finally, porcine GCs were washed thrice for 5 min each time, incubated in 10 μg/mL Hoechst 33,342 for 10 min, and then washed thrice with PBS. The cells were pictured under the fluorescence microscope (EVOS FL Atuo, Waltham, MA, USA), and the fluorescence intensity was analyzed using the ImageJ software (Home page of the software: https://imagej.net/software/fiji/downloads, Accessed date: 28 September 2020), National Institutes of Health, Bethesda, MD, USA).

### 2.3. RNA Isolation, Reverse Transcription, and Quantitative RT-PCR

After the treated Porcine GCs were washed with PBS three times, the total RNA was lysed from porcine GCs using Trizol reagent (Vazyme, Nanjing, China), followed by further experiments or storage at −80 °C, and then the total RNA was extracted by chloroform, isopropanol, and alcohol. The total RNA was synthesized using the HiScript II 1st strand cDNA synthesis Kit (Vazyme, Nanjing, China). Finally, the synthesized cDNA samples were tested using the qRT-PCR technique and the ChamQ Universal SYBR qPCR Master Mix (Vazyme, Nanjing, China). The primer sequences of this experiment are displayed in the Table 1 and the expression of the genes was normalized to that of the internal control Actin. All experiments satisfied three biological replicates. The experimental data of qRT-PCR were analyzed using 2^−ΔΔCt^.

### 2.4. Annexin V-FITC/PI Staining and Flow Cytometry

All groups of porcine GCs treated with or without AST were cultured to logarithmic cells, cleaned in PBS, digested, inoculated in six-well plates with Try, and collected in 1.5 mL EP tubes. According to the manufacturer’s instructions, the porcine GCs were examined using the Annexin V-FITC/PI Apoptosis Detection Kit (MULIT SCIENCES, Hangzhou, China). Each experiment was divided into black groups, Annexin V groups, PI groups, and Annexin V and PI groups. Subsequently, the grouped porcine GCs stained with Annexin V and PI, respectively, were mixed gently with a pipette gun and incubated at room temperature for 15 min, and flow cytometry was used for machine detection within 1 h. All experiment data were analyzed using FlowJo^TM^ v10.10 software (BD Sciences, Shanghai, China).

### 2.5. ROS Assay

The porcine GCs’ ROS levels were measured using an ROS Test Kit (Beyotime Biotechnology Inc., Shanghai, China). The cultured porcine GCs were treated with 10 μmol/L dichlorofluorescein diacetate (DCFH-DA; fluorescent probe), incubated at 37 °C for 30 min, and washed with serum-free medium or DMEM, and photos were taken with a confocal fluorescence microscope (Olympus, Tokyo, Japan, FV3000) at constant settings. The mean optical density values (IntDen/Area) were analyzed using ImageJ software (National Institutes of Health).

### 2.6. Determination of the Mitochondrial Membrane Potential

The porcine CGs’ mitochondrial membrane potential levels were measured using the Enhanced mitochondrial membrane potential assay kit with JC-1 (Beyotime Biotechnology Inc.). According to the instructions, the JC-1 dyeing solution was prepared, and the cultured porcine GCs treated with AX or without AX were added to DMEM and JC-1 dye solution as 1:1 into the six-well plate, mixed thoroughly, and incubated in the incubator at 37 °C for 30 min. The color of the dye changes from green (cytoplasmic JC-1 monomer) to red (mitochondrial JC-1 aggregates) as the mitochondrial membrane becomes more polarized. After incubation, the incubated porcine GCs were removed from the supernatant, cleaned with JC-1 dye buffer twice, added to 2 mL DMEM, and pictured under a confocal fluorescence microscope (Olympus, FV3000) at constant settings. The mean optical density values (IntDen/Area) were analyzed using ImageJ software (National Institutes of Health).

### 2.7. ELISA for Estrogen and Progesterone Levels

Estrogen and progesterone were detected by a Quickey pro porcine estradiol (E2) ELISA kit and Quickey pro porcine progesterone (P4) ELISA kit. A 1.5 mL sterile EP tube was used, and the cell culture solution was collected and centrifuged at 3000 rpm for 20 min at room temperature. According to the product instructions, standard wells, blank wells, and sample wells set up, 50 μL of different concentrations of standards was added to each standard well, and no samples or enzyme standard reagents were added to the blank wells. In the sample wells, each well was given 40 μL of the sample dilution, 10 μL of the sample to be tested, and 100 μL of the enzyme standard reagent in each empty well except the blank wells. The plate was sealed with a sealing film and incubated in the incubator at 37 °C for 1 h; then, the sealing film was removed and discarded liquid was shaken dry as much as possible, washing solution was injected into each well and left for 30 s, the liquid was discarded, this was repeated 5 times, and they were shook dry. Each well was given 50 μL chromogen A and 50 μL chromogen B, was shaken gently, and developed at 37 °C for 15 min under protection from light. At the end of the color development, each well was given 50 μL of the termination solution, and the absorbance was detected by a microplate reader within 15 min.

### 2.8. EdU Staining Proliferation

Granulose cell proliferation was detected by an EdU staining proliferation kit (RIBOBIO, Guangzhou, China). The EdU staining proliferation-related solutions were prepared according to the product instructions. The EdU solution (Reagent A) was diluted with the cell complete medium at a ratio of 1000:1. In total, 100 μL of 50 μM EdU medium was incubated into 96-well plates for 2 h. The medium was discarded, and PBS was added to wash twice for 5 min each. Each well given 50 μL PFA was incubated at room temperature for 30 min and discarded PFA. In each well, 50 μL 2 mg/mL glycine solution was added, incubated on a shaker for 5 min, and discarded glycine. Each well was given 100 μL PBS and incubated for 5 min on a shaker, and PBS was discarded. Then, 100 μL permeate was added to each well, incubated for 10 min on a shaker, and washed once for 5 min with PBS. Each well was given 100 μL of 1 × Apollo staining reaction solution, protected from light, and incubated at room temperature for 30 min, and the reaction solution was discarded. Thereafter, each well was given 100 μL permeate, and the shaker was washed three times for 10 min each time to remove the permeate. Then, each well given 10 μg/mL Hoechst 33,342 was incubated for 15 min. The cells were pictured under the fluorescence microscope (EVOS FL Atuo), and cell numbers were analyzed with ImageJ software (Home page of the software: https://imagej.net/software/fiji/downloads, Accessed date: 28 September 2020, National Institutes of Health).

### 2.9. Statistical Analysis

All data were from three replicates and are presented as the mean ± SEM unless particularly indicated. The statistics indicate an analysis of each set of data. Probability values (*p* values) less than 0.05 were considered to be significant.

## 3. Result

### 3.1. Isolation, Culture, and Identification of Porcine GCs

**Figure 1:**
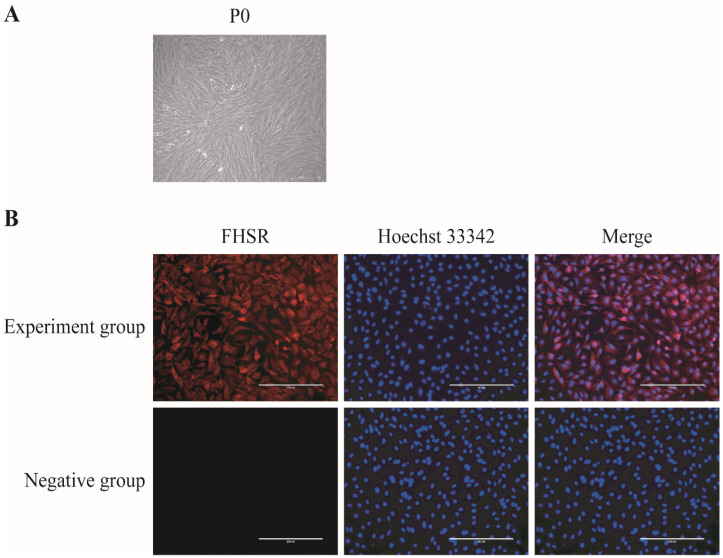
Isolation, culture, and identification of porcine GCs. (**A**) P0 generation granulosa cells were collected from porcine ovarian follicles for in vitro culture. Scale bar: 100 μm (**B**) Immunofluorescence staining for the granulosa cell-specific receptor FSHR was performed using the specific anti-FSHR for labeling in the control group. Scale bar: 200 μm.

Porcine granulosa cells are the object of this research study, which was intended to investigate the effect of being cultured in vitro on their physiological state. In our research, porcine primary granulosa cells (P0) isolated and cultured from ovarian follicles showed a small, clear outline as well as a fibroblast-like morphology (Figure 1A).

To further verify the isolated and cultured granulosa cells, cellular immunofluorescence staining was performed using the specific receptor protein FSHR on the GCs, and FSHR was expressed on the primary cell membrane of porcine GCs, as observed by fluorescence microscopy (Figure 1B). More than 90% of the isolated and cultured porcine granulocytes expressed FSHR protein, indicating the high purity of the porcine GCs isolated and cultured from the ovarian follicles. The cells could be used for subsequent experiments.

### 3.2. Effect of In Vitro Subculture of Porcine GCs on Their Morphology and Apoptosis

**Figure 2:**
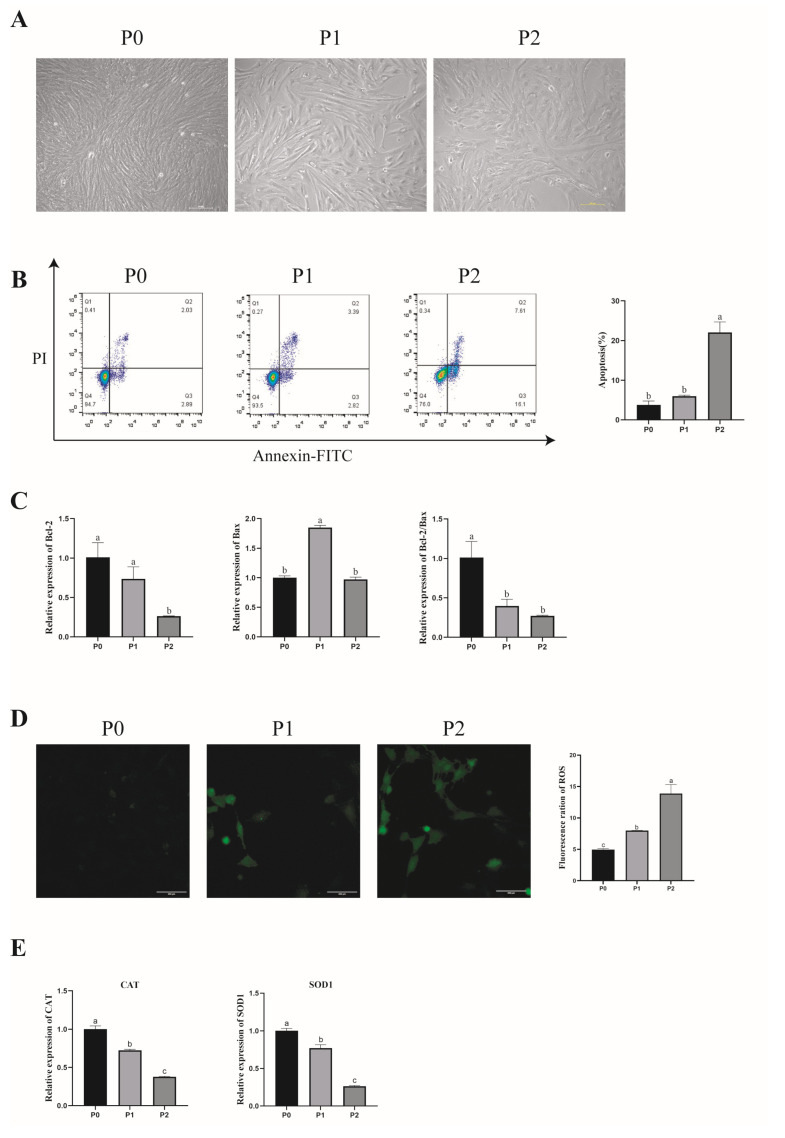
Effect of in vitro subculture of porcine GCs on their morphology, apoptosis, and antioxidant capacity. (**A**) Morphological changes in porcine GCs in different passages in vitro. Scale bar: 100 μm. (**B**) Apoptosis rate for three passages of porcine GCs. Values: mean ± SEM; one-way ANOVA was used. (**C**) Expression of apoptotic genes (Bcl-2, Bax, and Bcl-2/Bax). (**D**) Differences in ROS levels in three passages of porcine granulosa cells. Values: mean ± SEM; one-way ANOVA was used. Scale bar: 200 μm. (**E**) Expression of Anti-oxidation-related genes (CAT, SOD1). Values: mean ± SEM; one-way ANOVA was used. P0: Primary porcine GCs cultured in vitro. P1: porcine GCs after the first passage were cultured in vitro. P2: porcine GCs after the second passage were cultured in vitro. Values with different letters are significantly different (*p* < 0.05).

The purpose of this study was to explore the effect of in vitro culture on the physiological state of porcine GCs. We first observed the morphology of different porcine GCs passages using a microscope. The results showed that P0 had a small and clear outline; the first passage (P1) had an increased size and its outline was blurred; the second passage’s (P2) cell volume was increased even more, it had more tentacles, its outline was blurred, and it had a polygon morphology (Figure 2A).

When the number of passages increased and the morphology was changed, we further detected the apoptosis and ROS levels to estimate whether the physiological state would change. First, the apoptosis of porcine GCs was analyzed by flow cytometry. The flow cytometry results showed that the apoptosis rate of P2 was significantly higher than in P0 and P1 (*p* < 0.05). (Figure 2B). In addition, B-cell lymphoma 2 (*Bcl-2*) mRNA expression was significantly higher in P0 than in P2, and that in P0 was higher than in P1, but the difference was not significant (*p* > 0.05). The expression level of apoptosis regulator Bax (*Bax*) mRNA expression was significantly higher in P1 than in P0 and P2 (*p* < 0.05). The apoptosis rate ratio (*Bcl-2/Bax*) mRNA expression levels of P1 and P2 were significantly lower than in P0 (*p* < 0.05), and those in P2 were lower than in P1, but the difference was not significant (*p* > 0.05) (Figure 2C). These results indicate that the apoptotic rate of porcine GCs increases with the number of passages.

### 3.3. Effect of In Vitro Subculture of Porcine GCs on Antioxidant Capacity

To explore the effect of different passages on ROS production in porcine GCs, we used a fluorescent probe DCFH-DA to detect ROS levels. The result showed that ROS levels significantly increased with cultured passages (*p* < 0.05). (Figure 2D). At the same time, the mRNA expression of antioxidant genes significantly decreased with cultured passages, including Catalase (*CAT*) and superoxide dismutase 1 (*SOD1*) (*p* < 0.05) (Figure 2E).

### 3.4. Effects of Different Concentrations of Astaxanthin on the Morphology and Viability of Porcine GCs

**Figure 3:**
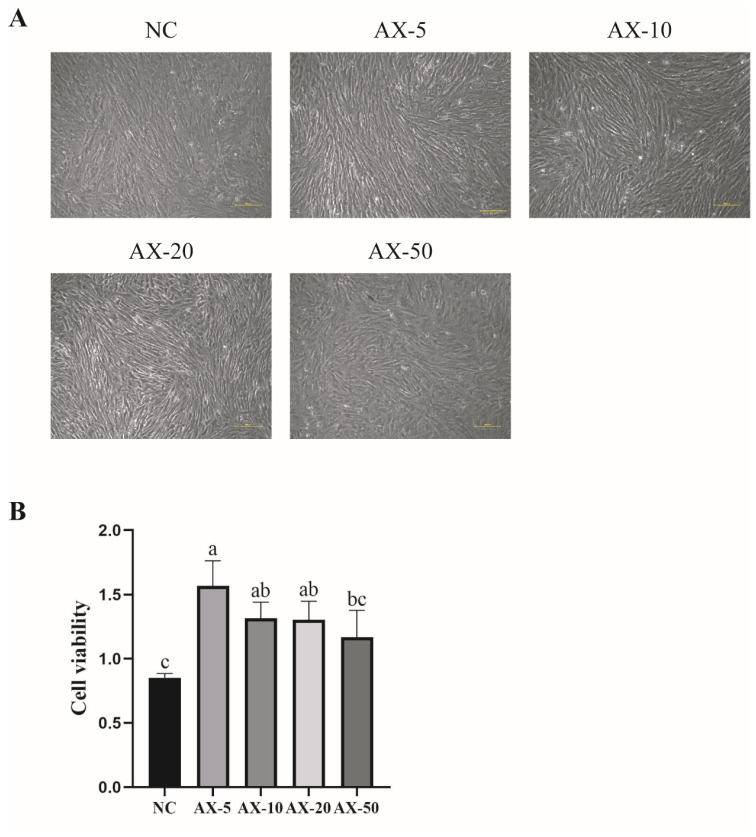
Effects of different concentrations of astaxanthin on the morphology and viability of porcine GCs. (**A**) Morphological changes in porcine GCs supplemented with astaxanthin at different concentrations. Scale bar: 100 μm. (**B**) The difference in granulosa cell activity in porcine with different concentrations of astaxanthin. Values: mean ± SEM; one-way ANOVA was used. Values with different letters are significantly different (*p* < 0.05). NC: Control group; astaxanthin was not added. AX-5: Treatment group; 5 μmol/L astaxanthin was added into the cell medium. AX-10: Treatment group; 10 μmol/L astaxanthin was added to the cell medium. AX-20: Treatment group; 20 μmol/L astaxanthin was added to the cell medium. AX-50: Treatment group; 50 μmol/L astaxanthin was added to the cell medium.

Our experiment showed that porcine GCs’ oxidative damage increased during cultured passages in vitro; AST, as a strong antioxidant, can effectively reduce this. We used a microscope and cck-8 kit to detect the morphology and cell viability of the cultured porcine GCs in vitro. The results showed that, for the morphology in the NC group, the AX-5 and AX-10 were small, there was a clear outline, and there was a fibroblast-like morphology. The AX-20 began to grow larger, it flattened, and the outline began to blur, and when the astaxanthin concentration increased to 50 μmol/L, the cell volume became larger, the outline was blurred, the tentacles increased, and the cells were polygonal (Figure 3A). In addition, the cell viability in the AX-5 was significantly higher than in the NC group and in AX-50 (*p* < 0.05), but it was similar to that of other groups (*p* > 0.05) (Figure 3B).

### 3.5. Effect of Adding Different Concentrations of Astaxanthin on the Proliferation of Porcine GCs

**Figure 4:**
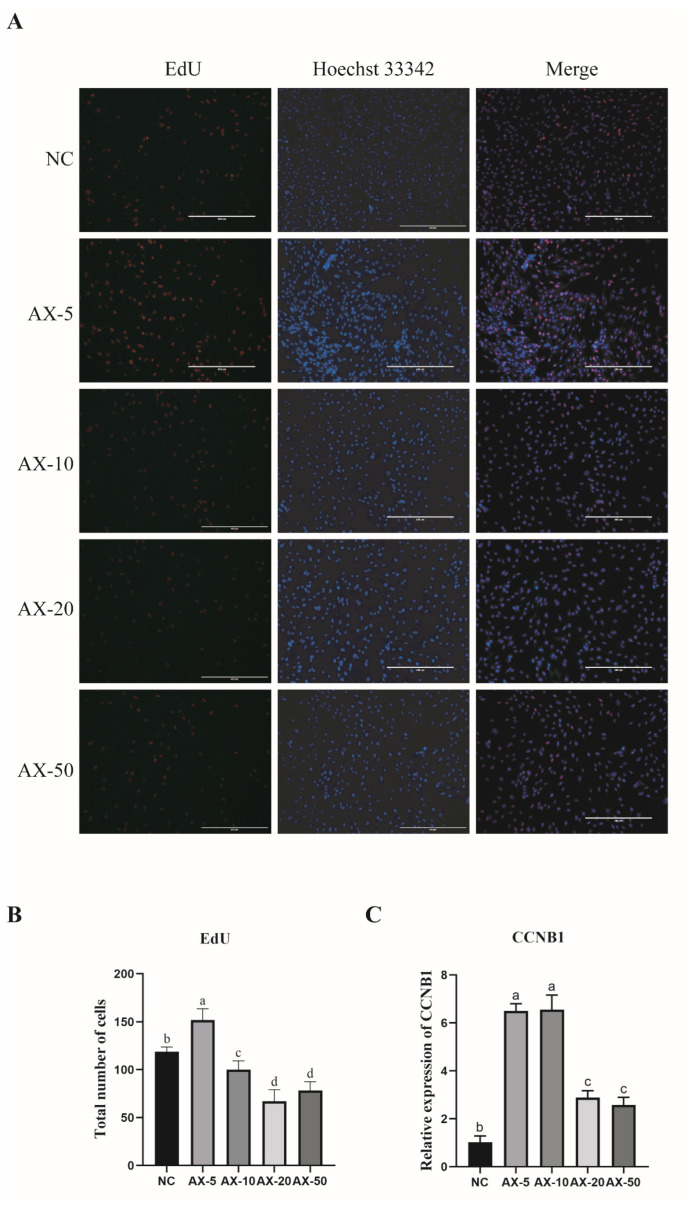
Effect of adding different concentrations of AST on the proliferation of porcine GCs. (**A**) EdU detection of PGC proliferation fluorescence. Scale bar: 400 μm. (**B**) The total number of cells that proliferate. Values: mean ± SEM; one-way ANOVA was used. (**C**) Expression of genes (CCNB1: Cyclin B1). Values: mean ± SEM; one-way ANOVA was used. Values with different letters are significantly different (*p* < 0.05).

The results showed that adding AST could improve the morphology and enhance the cell viability of porcine GCs. To further explore the effect on porcine GCs proliferation of added AST, we used the effect of different concentrations of AST on cell proliferation via the EdU kit. The results showed that the AX-5 was significantly higher than in other treatment groups (Figure 4A,B). The AX-5 of the mitotic-specific cyclin-B1 (CCNB1) mRNA expression was similar to the AX-10 (*p* > 0.05) and significantly higher than in other groups (*p* < 0.05) (Figure 4C). These data indicate that the AX-5 could increase the proliferation capacity of porcine GCs.

### 3.6. Effect of Different Concentrations of AST on Apoptosis of Porcine GCs

**Figure 5:**
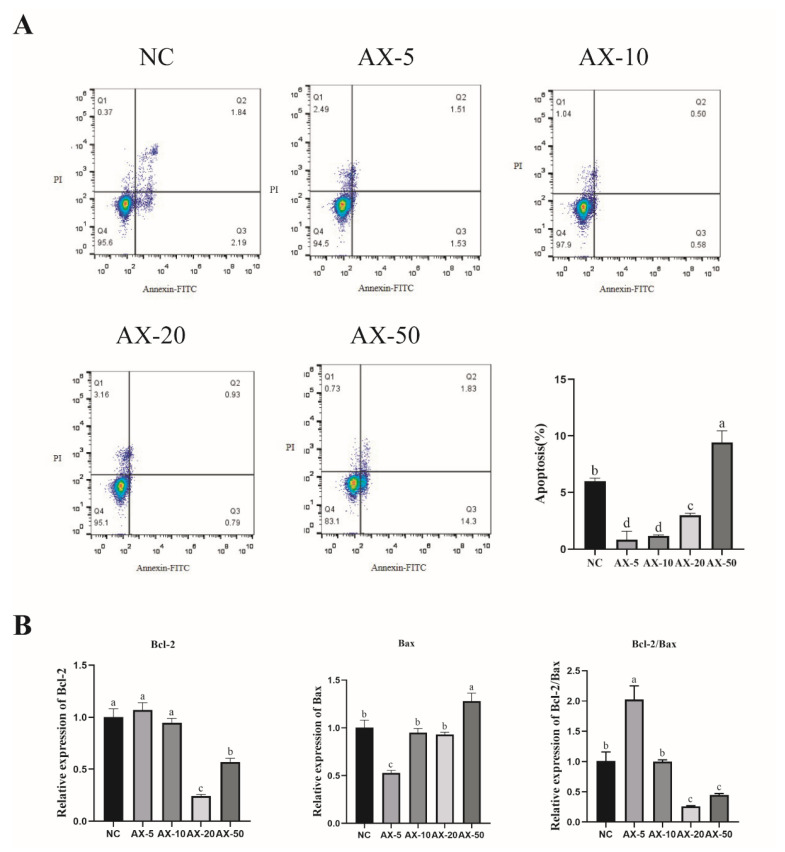
Effect of different concentrations of AST on the apoptosis of porcine GCs. (**A**) Apoptosis rate of porcine GCs treated with different concentrations of AST. Values: mean ± SEM; one-way ANOVA was used. (**B**) Expression of apoptotic genes (Bcl-2, Bax, and Bcl-2/Bax). Values: mean ± SEM; one-way ANOVA was used. Values with different letters are significantly different (*p* < 0.05).

Astaxanthin has antioxidant, anti-apoptotic, and anti-inflammatory functions. In measuring the different concentration of AST of porcine GCs by flow cytometry, this in vitro study showed that the AX-5 was similar to AX-10 (*p* > 0.05) and significantly lower than other groups (Figure 5A), indicating the alleviation of porcine GCs’ apoptosis when astaxanthin is added. To further determine whether porcine GCs’ apoptosis was alleviated while cultured in vitro, we compared the ratio of *Bcl-2/Bax* to verify the changes in the expression levels of porcine GCs’ apoptosis-related genes. RT-PCR showed that the AX-5 *Bcl-2* mRNA expression was similar to that of the NC and AX-10 groups (*p* > 0.05) and significantly higher in the other groups (*p* < 0.05); Bax mRNA expression was significantly lower in the other treatment groups (*p* < 0.05); the Bcl-2/Bax ratio was significantly higher in the other groups (*p* < 0.05) (Figure 5B).

### 3.7. Effect of Different Concentrations of Astaxanthin on the Antioxidant Properties of Porcine GCs

**Figure 6:**
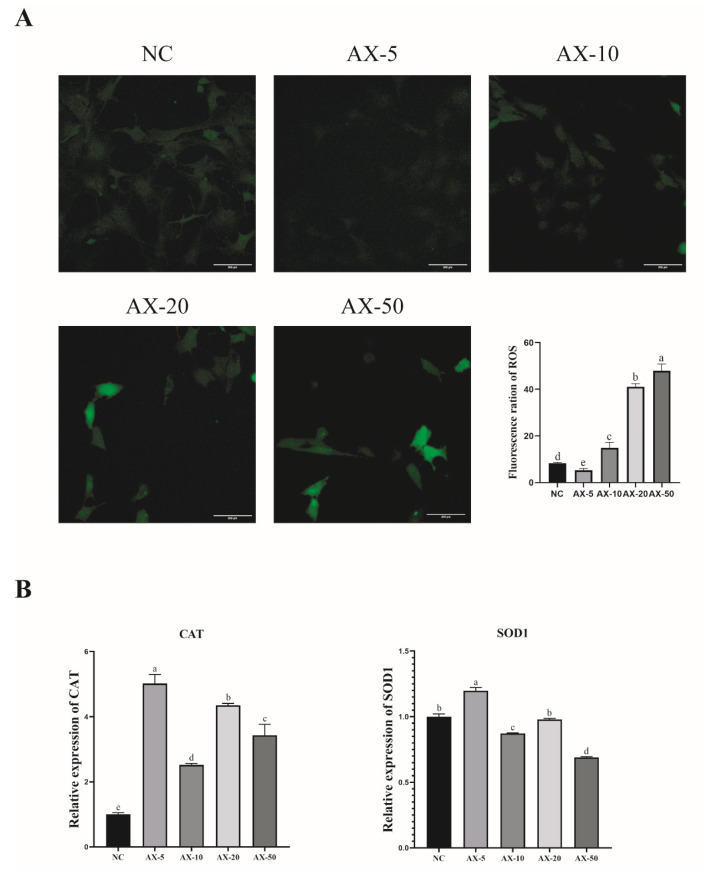
Effect of different concentrations of astaxanthin on ROS and antioxidant genes of porcine GCs. (**A**) Effects of astaxanthin supplementation with different concentrations on the ROS of porcine GCs. Values: mean ± SEM; Scale bar: 200 μm. (**B**) Expression of anti-oxidation-related genes (CAT, SOD1). Values: mean ± SEM; one-way ANOVA was used. Values with different letters are significantly different (*p* < 0.05).

During the experimental study, we found that the ROS levels of porcine GCs significantly increased while being cultured in vitro. We added different concentrations of AST into the medium to verify whether AST would reduce the ROS level. The AX-5 group showed significantly lower ROS fluorescence intensity compared to the other treatment groups (*p* < 0.05) (Figure 6A). The effect of AST on antioxidants in porcine GCs was researched. After the porcine GCs were treated with AST, the AX-5 mRNA expression of antioxidant genes was significantly increased in the porcine GCs, including *CAT* and *SOD1* (Figure 6B). In summary, the AX-5 group can promote the viability of porcine GCs, improve the proliferation ability, reduce intracellular ROS levels, and inhibit apoptosis. Consequently, subsequent experiments selected 5 μmol/L as the AST treatment group.

### 3.8. Effect of Astaxanthin on the Mitochondrial Function of Porcine GCs

**Figure 7:**
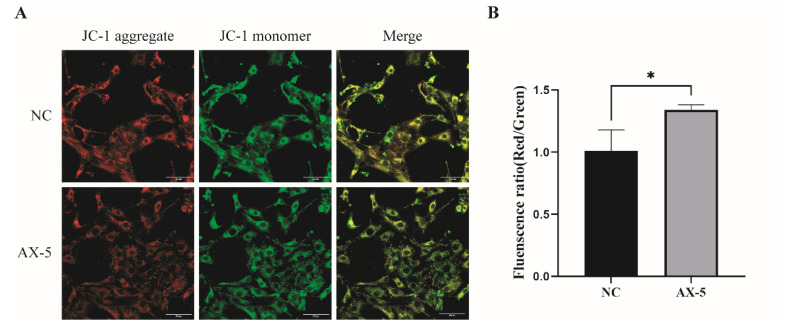
Effect of astaxanthin on mitochondrial function in porcine GCs. (**A**) Stained fluorescent picture with JC-1. Scale bar = 200 μm; (**B**) The mitochondrial membrane potential was analyzed by the Red/Green fluorescence ratio; a paired samples *t*-test was used. Asterisks indicate statistical significance between the two groups (* *p* < 0.05).

Mitochondrial membrane potential is a basic component of maintaining electrochemical potential during ATP synthesis. Our experiment found porcine GCs’ physiological state. We tested whether AST could improve mitochondrial activity and stabilize the state of porcine GCs in vitro. Then, the research measured the mitochondrial membrane potential of porcine GCs using the fluorescent probe JC-1 (Figure 7A). The red/green JC-1 signal ratio in the 5 μmol/L AST treatment group was significantly higher than that of other treatment groups (*p* < 0.05, Figure 7A), indicating that the AX-5 group improved mitochondrial activity and stabilized the physiological state of porcine ovarian granulose cells cultured in vitro.

### 3.9. Hormone Levels in the Astaxanthin of Porcine GCs

**Figure 8:**
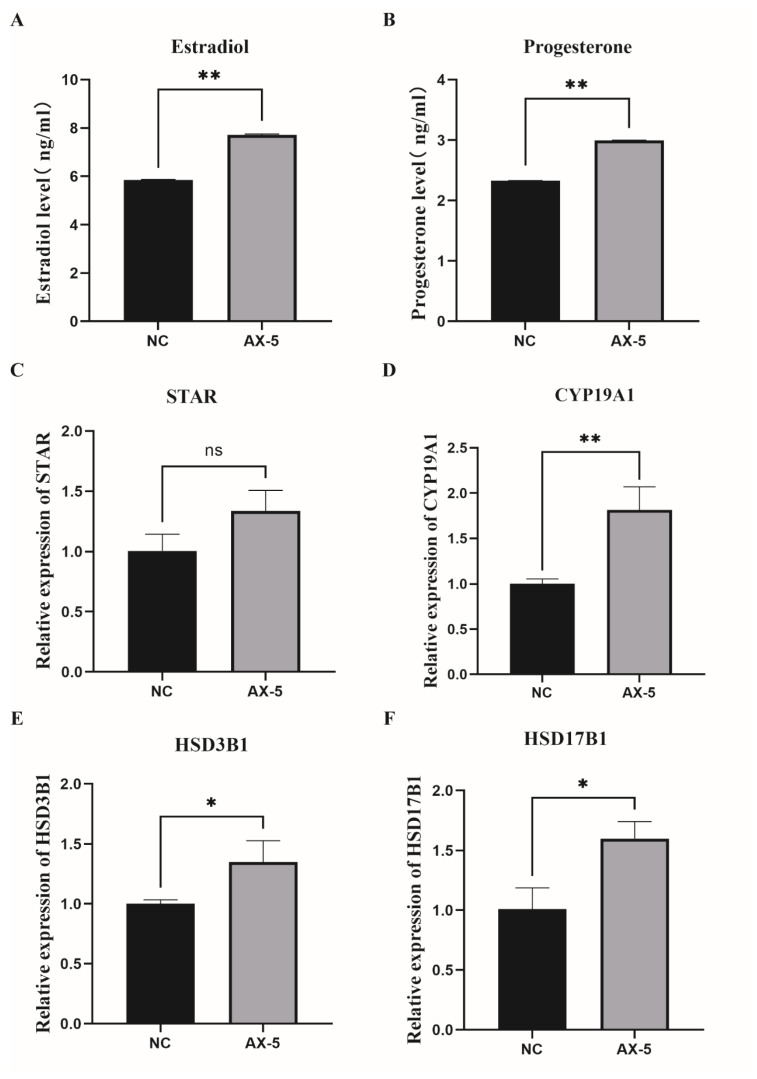
Hormone levels in the astaxanthin of porcine GCs. (**A**) The concentrations of estradiol in porcine GCs treated with astaxanthin. (**B**) The concentrations of progesterone in porcine GCs treated with astaxanthin. (**C**–**F**) E2 and P4 synthesis-related genes (*STAR*, *CYP19A1*, *HSD3B1*, *HSD17B1*) were expressed in porcine GCs treated with astaxanthin. All represent the mean ± SEM; a paired samples *t*-test was used. Asterisks indicate statistical significance between the two groups (ns: *p* > 0.05, * *p* < 0.05, ** *p* < 0.01).

GCs regulate follicular development and oocyte maturation by secreting hormones. We treated porcine GCs with a 5 μmol/L concentration. We exploited the ELISA detection kit to measure the concentrations of estradiol (E2) and progesterone (P4) in the culture medium, respectively, and those in the AX-5 group were significantly higher than those in the NC group of E2 (*p* < 0.05, Figure 8A); those of the AX-5 group were significantly higher than those in the NC group of P4 (*p* < 0.05). (Figure 8B). Our experiments found that astaxanthin played a positive role in porcine GCs cultured in vitro. After we tested hormone-related genes by qRT-PCR analysis, the mRNA expression showed that the AX-5 group was significantly higher than in the NC group (*p* < 0.05, Figure 8C,D), including steroidogenic Acute Regulatory Protein (STAR) and cytochrome P450 Family 19 subfamily A Member1 (CYP19A1). Hydroxy-Delta-5-Steroid Dehydrogenase and 3 Beta-And Steroid Delta-lsomerase1 (HSD3B1) and Hydroxysteroid 17-Beta Dehydrogenase1 (HSD17B1) mRNA expression were also significantly increased after 5 μmol/L astaxanthin treatment (*p* < 0.05, Figure 8E,F). These results showed that astaxanthin treatment could promote the synthesis and secretion of hormone and hormone-related genes expression.

## 4. Discussion

The current study indicates that granulosa cells are involved in maintaining the basic physiological functions of ovarian follicles and provide the necessary nutrients and microenvironment for oocytes [23,24]. Our study found porcine GCs’ morphological changes, apoptosis, and oxidative damage while being sub-cultured in vitro. AST, as a strong antioxidant, can effectively clean ROS. However, the physiological state of cultured porcine GCs can be alleviated by adding AST with an appropriate concentration and maintaining the essential physiological function of GCs. Thus, the purpose of the study is to explore whether AST could alleviate the effect of oxidative stress on cultured porcine GCs in vitro by adding exogenous AST.

Follicular development and oocyte maturation can hardly be accomplished without granulosa cells’ proliferation and the differentiation, synthesis, and secretion of estrogen, as well as essential growth factors [25,26]. The in vitro fertilization of bovine oocytes with GCs can significantly increase the cleavage rate and blastocyst rate of bovine embryos and decrease the abnormal fertilization rate [27]. Further research determined that an analysis of porcine GCs’ gene expression profiles showed a positive correlation with embryo quality, suggesting that GCs could be used to predict embryonic development potential [28]. Therefore, this study collected porcine GCs as the research object to further explore the influence of cultured porcine GCs in vitro on the physiological state. We successfully collected and cultured porcine GCs from porcine ovary follicles. Then, our experiment used an anti-FSHR antibody to examine porcine GCs, and the result showed that most of the collected and cultured porcine GCs expressed the FSH protein. This experimental method could be used to collect porcine GCs in subsequent experiments.

In bovine granulosa cells (GCs) cultured in vitro, senescent characteristics, including nuclear area enlargement, reduced proliferation, and cell cycle arrest, have been observed with increasing culture passages [29]. In this study, porcine GCs exhibited cell area expansion and decreased proliferation with increased in vitro culture passage. Morphological changes from the follicular phase to the luteal phase in GCs are characterized by an increased cell membrane area and breakdown in the late stages of apoptosis [30]. Microscopic observations revealed more pronounced morphological changes in passage 2 (P2) than in passage 1 (P1), including an increased cell area, a decreased fibroblast-like morphology, and flattened cells. GCs transition from squamous and cuboidal shapes to cuboidal forms, forming layers around the oocyte during follicular development [31,32]. We found that cells in the second passage and subsequent passages exhibited severe morphological deformation and reduced proliferation and were more susceptible to contamination during in vitro culture experiments. Therefore, this research selected first-passage cells for subsequent experiments.

GCs are essential for follicle initiation and development. Research indicates that oxidative stress occurs when the balance between ROS production and antioxidant system regulation is disturbed, leading to elevated ROS levels, apoptosis, and mitochondrial dysfunction [33]. The study of Mojtaba Eslami et al. examined the fact that AST inhibited ROS generation and protected human GCs against oxidative stress [34]. Our findings showed that oxidative stress increased in porcine GCs during the in vitro subculture, as evidenced by elevated ROS levels and apoptosis. Apoptosis-related gene expression (*BAX*, *Bcl-2*) indicated increased apoptosis levels, while antioxidant-related gene expression (*CAT*, *SOD1*) suggested decreased antioxidant capacity in GCs across different passages. Therefore, we investigated the effect of astaxanthin (AST) supplementation on GCs during the in vitro culture. AST is a natural C40 carotenoid with numerous reported antioxidant and anti-inflammatory activity [35,36]. It has been shown to have 100–500-times-higher antioxidant capacity than other antioxidants [37,38]. Previous studies have reported that human GCs’ total antioxidant capacity in the AST treatment group significantly increased, as well as the protective role of AST against oxidative stress [34,39]. Our study discovered that the proliferation levels in the low-concentration group astaxanthin of in vitro cultured porcine GCs were promoted, but those of the high-concentration group were inhibited. The mRNA expression level of proliferation-related gene CCNB is consistent with the EdU assay results. 

Furthermore, follicular atresia is primarily indicated by GC apoptosis [40]. According to the study by Maryam et al., AST could induce the apoptosis of LS-80 cells by increasing the expression of apoptosis-related genes [41]. Researchers investigated whether BPA + AST could significantly improve antioxidant genes and anti-apoptotic genes in human oocytes [42]. Therefore, adding 5 μmol/L AST is an effective method for decreasing the porcine GCs apoptosis rate in this study; the 5 μmol/L AST treatment group apoptosis rate reduced, and the apoptosis-related genes’ mRNA expression levels were significantly lower than in the control group. ROS formation can induce oxidative stress and then lead to the accumulation of ROS-related damage in DNA, proteins, and lipids and may cause progressive cellular dysfunction and, ultimately, apoptosis [43,44]. Astaxanthin, one of the synthetic antioxidants, can effectively protect against damage caused by ROS, which is an endogenous oxidative stress response when oxidative stress increases [45]. The research results of Jun Qiang et al. showed that adding appropriate concentrations of astaxanthin to the feed of Nile tilapia can effectively its the level of apoptosis of its granulosa cells, alleviate the oxidative stress of ovarian tissue, and improve oocyte development potential [46]. Researchers’ studies have shown that the addition of astaxanthin to different species can effectively alleviate cellular oxidative damage and protect against oxidative stress [21,34,47]. To determine oxidative stress, the formation of ROS as well as the antioxidant defense potential need to be measured, and antioxidant-related genes’ mRNA expression needs to be examined by the qPCR method. This study found that the 5 μmol/L AST treatment group can decrease ROS levels as well as increase antioxidant-related genes’ mRNA expression, including CAT and SOD1. We found that the 20 μmol/L AST treatment group or other high concentrations induced as rise in ROS levels. Thereby, appropriate antioxidant supplementation may benefit this important physiological balance between ROS formation and neutralization.

Mitochondria, forming the cellular energy production center, are also a source of ROS. Research has shown that AST can prevent stress-induced mitochondrial dysfunction, increasing the mitochondrial membrane potential (MMP), mitochondrial content, and ATP production and reducing the mitochondrial ROS production [48,49]. In this study, we found that 5 μmol/L AST treatment increased MMP in porcine GCs, as detected by MMP assay kits. 

Additionally, we evaluated steroid hormone synthesis and secretion in porcine GCs. The 5 μmol/L AST treatment group showed significantly higher E2 and P4 synthesis and secretion compared to the control group. Steroid hormones can affect GC growth and development and follicular fluid formation, involving proliferation, apoptosis, and oxidative damage [50]. Xiao-Wei Li et al. demonstrated ATR-induced mitochondrial dysfunction and steroid disorders in GCs [51]. E2 and P4 are synthesized through a multistep biochemical reaction that converts cholesterol into steroid hormones involving multiple metabolic enzymes. STAR is a rate-limiting enzyme, and the HSD family catalyzes P4 production, converting a small amount to testosterone, which is then converted to E2 by CYP19A1 aromatase [52,53,54]. The study by Weizhao He et al. determined that astaxanthin supplementation could improve the antioxidant capacity and promote the production of reproductive hormones of laying hens’ follicular granulosa cells [55]. We found that the 5 μmol/L AST treatment group promotes the mRNA expression of steroid hormone-related genes (STAR, CYP19A1, HSD3B1, HSD17B1). These results indicated that AST can enhance E2 and P4 synthetic and related hormone levels in porcine GCs and promote the porcine GCs state cultured in vitro, which lacked hormones.

## 5. Conclusions

In summary, the supplementation of 5 μmol/L astaxanthin in porcine GCs cultured in vitro resulted in enhanced cell viability and proliferation, decreased levels of reactive oxygen species, reduced apoptosis, increased levels of mitochondrial membrane potential, and an increased synthesis and secretion of steroid hormones. Astaxanthin also upregulated the expression of genes related to antioxidants and steroid hormones, thereby enhancing the antioxidant function in porcine granulosa cells. Consequently, astaxanthin can serve as a potent antioxidant supplement for in vitro culture, improving the physiological state of porcine granulosa cells.

## Figures and Tables

**Table 1:** Quantitative real-time PCR primers of genes.

Genes	Primer Sequences	Length (bp)	Tm (°C)	Accession No.
*β-actin*	F: GATGACGATATTGCTGCGCT R: TTCTCCATGTCGTCCCAGTT	248	60	XM_021086047.1
*Bcl-2*	F: TGAGTTCGGTGGGGTCATGT R: GGCCCATACAGCTCCACAAAG	159	60	XM_021099593
*Bax*	F: GGCCCTTTTGCTTCAGGGTTT R: GACACTCGCTCAACTTCTTGG	119	60	XM_003127290.5
*CCNB1*	F: CATCAATTACCTGGACCGCT R: CTGAGGCTTGATGGAGTTGT	163	60	NM_001170768.1
*SOD1*	F: ATTCTGTGATCGCCCTCTCG R: ACTTCCAGCATTTCCCGTCT	125	60	NM_001190422.1
*CAT*	F: AGATGAAGCATTGGAAGGAGC R: TCTCAGGAATTCTCTCCCGGT	162	60	NM_214301.2
*STAR*	F: AAAGTGATCCCTGACGTGGG R: CGTGAGTGATGACCGTGTCT	175	60	NM_213755.2
*CYP19A1*	F: GGCTATGTGGACGTGTTGACC R: TGAGAAGGAGAGCTTGCCATG	164	60	NM_214429.1
*HSD3B1*	F: CAGCCAGGTATGGCCGAC R: CGGACTACATGTTCCCCCAG	89	60	NM_001004049.2
*HSD17B1*	F: AGTCCTTGGCTTACCAACCG R: TTCTGCATTGGAACCCCTCC	177	60	NM_001128472.1

## Data Availability

Data is contained within the article.
